# Molecular assessment of *voltage-gated sodium channel* (*VGSC*) gene mutations in *Rhipicephalus microplus* from Guangxi, China

**DOI:** 10.1186/s13071-024-06383-6

**Published:** 2024-07-17

**Authors:** Na Jiang, Ting Xie, Chunfu Li, Rui Ma, Ai Gao, Mengyun Liu, Shurong Wang, Qingan Zhou, Xiankai Wei, Jian Li, Wei Hu, Xinyu Feng

**Affiliations:** 1 College of Life Sciences, Inner Mongolia University, Hohhot, China; 2 Hechi Animal Disease Prevention and Control Center, Hechi, Guangxi China; 3 Guangxi Center for Animal Disease Control and Prevention, Nanning, Guangxi China; 4 Basic Medical College, Guangxi University of Chinese Medicine, Nanning, Guangxi China; 5 Department of Infectious Diseases, Huashan Hospital, State Key Laboratory of Genetic Engineering, Ministry of Education Key Laboratory for Biodiversity Science and Ecological Engineering, Ministry of Education Key Laboratory of Contemporary Anthropology, School of Life Sciences, Fudan University, Shanghai, China; 6 School of Global Health, Chinese Center for Tropical Diseases Research, Shanghai Jiao Tong University School of Medicine, Shanghai, China; 7 One Health Center, Shanghai Jiao Tong University-The University of Edinburgh, Shanghai, 20025 China

**Keywords:** Guangxi, *Rhipicephalus microplus*, Pyrethroids, *VGSC*, Acaricide resistance

## Abstract

**Background:**

Pyrethroid chemicals are one of the main acaricides used against ticks. Resistance to these chemicals has been reported to be associated with mutations in the *voltage-gated sodium channel* (*VGSC*) gene of the *Rhipicephalus microplus*. This study investigates *R. microplus* resistance to pyrethroids in Guangxi region of China, marking one of the first research efforts in this area. The findings are intended to provide vital baseline for the effective implementation of localized tick control strategies.

**Methods:**

From March to July 2021, 447 *R. microplus* tick samples were collected from five prefecture-level cities in Guangxi. Allele-specific polymerase chain reaction (AS-PCR) was used to amplify segments C190A and G215T of the domain II S4-5 linker and T2134A of domain III S6 in the *VGSC*, to detect nucleotide mutations associated with resistance to pyrethroid acaricides. Subsequent analyses were conducted to ascertain the prevalence, types of mutations, and genotypic distributions within the sampled populations.

**Results:**

Mutations within *VGSC* gene were identified across all five studied populations of *R. microplus*, although the mutation rates remained generally low. Specifically, the most prevalent mutation was C190A, observed in 4.9% of the samples (22/447), followed by G215T at 4.0% (18/447), and T2134A at 1.3% (6/447). The distribution of mutations across three critical sites of the *VGSC* gene revealed four distinct mutation types: C190A, G215T, C190A + G215T, and T2134A. Notably, the single mutation C190A had the highest mutation frequency, accounting for 4.3%, and the C190A + G215T combination had the lowest, at only 0.7%. The analysis further identified seven genotypic combinations, with the wild-type combination C/C + G/G + T/T predominating at a frequency of 90.4%. Subsequently, the C/A + G/G + T/T combination was observed at a frequency of 4.3%, whereas the C/C + T/T + T/T combination exhibited the lowest frequency (0.2%). Additionally, no instances of simultaneous mutations at all three sites were detected. Geographical differences in mutation types were apparent. Both samples from Hechi to Chongzuo cities exhibited the same three mutation types; however, C190A was the most prevalent in Hechi, while G215T dominated in Chongzuo. In contrast, samples from Beihai to Guilin each exhibited only one mutation type: G215T occurred in 12.5% (4/32) of Beihai samples, and C190A in 7.5% (4/53) of Guilin samples.

**Conclusions:**

These findings underscore the relatively low frequency of *VGSC* gene mutations in *R. microplus* associated with pyrethroid resistance in the Guangxi, China. Moreover, the variation in mutation types and genotypic distributions across different locales highlights the need for regionalized strategies in monitoring and managing pyrethroid resistance in tick populations. This molecular surveillance is crucial for informing targeted control measures and mitigating the risk of widespread resistance emergence.

**Graphical Abstract:**

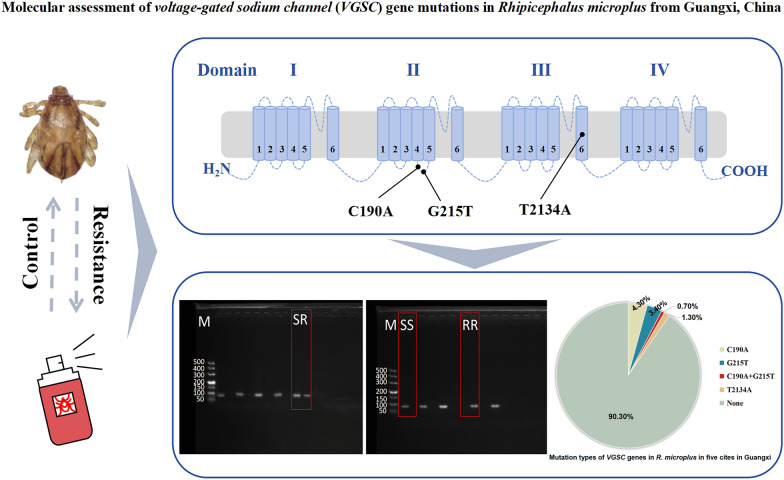

## Background

*Rhipicephalus* (*Boophilus*) *microplus* (Canestrini, 1887) (Acari: Ixodidae), a member of the hard tick family, primarily parasitizes large livestock such as cattle and goats. Due to its host preference and widespread global distribution, this tick species is considered the most economically significant ectoparasite of livestock [1–3]. *R. microplus* is a dominant tick species in the Guangxi Zhuang Autonomous Region of China, posing significant challenges to local food supply stability and safety, as well as to the income of farmers and the economic development of agricultural and pastoral areas [4, 5]. Historically, the use of chemical acaricides has been the main method for effective tick control. However, the extensive and prolonged use of these chemicals has led to the development of resistance among ticks, with resistance levels increasing in recent years, posing serious threats to environmental and food safety [6].

Traditional chemical acaricides include organochlorines, organophosphates, carbamates, synthetic pyrethroids, and ivermectin, with variations in the types of acaricides used in different regions depending on the dominant tick populations and climatic conditions [7, 8]. Synthetic pyrethroids, known for their safety and efficacy, have played a significant role in tick control. However, due to increased usage and frequency, resistance to synthetic pyrethroids in *R. microplus* was first reported in Australia in the late twentieth century [9]. Subsequently, resistance to pyrethroids has rapidly spread worldwide, presenting significant challenges to tick control efforts [10–13]. The resistance of *R. microplus* to synthetic pyrethroids is primarily attributed to mutations in the *voltage-gated sodium channel* (*VGSC*) gene [14, 15]. *VGSC* serves as the target site for pyrethroid acaricides, and mutations in this gene lead to structural changes in the sodium channel proteins, reducing the interaction between pyrethroids and their target, thereby diminishing the ticks’ sensitivity to these acaricides [16]. The most common mutation associated with pyrethroid resistance, C190A, occurs in the domain II S4-5 linker region of *R. microplus* [12, 17]. Other mutations in the domain II S4-5 linker region, such as G215T, T170C, G184C, and C148T, have also been identified [18–20]. Additionally, the T2134A mutation in domain III S6 of the *VGSC* gene has been found and is associated with pyrethroid resistance [21, 22].

Global research efforts into *R. microplus* have cataloged various mutations in the *VGSC* gene, including G215T, T170C, C189A, C2136A, C148T, G184C, and C190G, which are implicated in pyrethroid resistance [19, 20, 23, 24]. In our study, we specifically focused on the mutations C190A, G215T, and T2134A, as a thorough literature review identified these mutations as being significantly associated with enhanced pyrethroid resistance. Notably, available literature indicates that there has been virtually no research from China on this specific aspect. Given the critical role of *R. microplus* in the development of resistance to synthetic pyrethroids and its dominance in Guangxi, China, the results will aid in understanding the molecular basis of tick resistance in Guangxi and the potential risks of resistance, offering a scientific basis for developing effective local tick control strategies.

## Methods

### Sample collection and initial processing

Ticks were collected from hosts across defined sampling sites and deposited in individual vials with small holes in the cap to ensure adequate ventilation. Detailed information regarding the date, exact location of collection, type of host, and the specific site on the host from which the tick was removed was recorded for each specimen. Upon arrival at the laboratory, each sample was cataloged in a database before undergoing identification and processing. Ticks were identified to species level using standard taxonomic keys under a dissecting microscope.

Prior to storage, ticks underwent a cleaning process to remove adherents such as soil, feces, and animal hair, which is crucial for maintaining sample integrity. Each tick was initially soaked in distilled water for 1 min, then washed three times with 75% ethanol to eliminate residual bacteria on their surface, followed by a final rinse with distilled water. Only after these cleaning steps were the ticks transferred to a −80 °C refrigerator for cryopreservation, ensuring that samples were free of contaminants that could interfere with subsequent analyses.

### Tick genomic DNA preparation

Post-identification, ticks were grouped by their respective collection sites to facilitate comparative analyses. After their initial cleaning and before being stored, ticks were placed in 1.5 mL centrifuge tubes. A volume of 50 µL of phosphate buffered saline (PBS) buffer was added to each tube, and the tick tissues were disrupted into a suspension using a high-throughput tissue homogenizer (FAST-24, Thermo Fisher, USA). After centrifugation to collect the supernatant, DNA samples were prepared using the DNeasy Blood and Tissue Kit (QIAGEN, Germany) for the ticks to be tested.

### Molecular identification of ticks

In this study, tick species were identified based on the *COXI* gene sequence using the PCR method [25, 26]. The *COXI* fragment was amplified using synthesized forward (5′- GGTCAACAAATCATAAAGATATTGG-3′) and reverse (5′-TAAACTTCAGGGTGACCAAAAAATCA-3′) primers [26]. The PCR reaction volume was set at 25 μL, which included 1.5 μL of DNA sample as the template, 0.5 μL of each forward and reverse primer, 12.5 μL of 2× EasyTaq PCR SuperMix (2× EasyTaq^®^ PCR SuperMix, Transgen, Beijing, China), and 10 μL of ddH_2_O (double distilled water). The PCR was performed in a Veriti 96 Well Thermal Cycler (Applied Biosystems, Singapore) with the following cycling conditions: initial denaturation at 94 °C for 5 min, followed by 35 cycles of denaturation at 94 °C for 30 s, annealing at 48 °C for 30 s, extension at 72 °C for 60 s, and a final extension at 72 °C for 5 min. The PCR products were analyzed via 2% agarose gel electrophoresis. Based on the visualization results from the gel imaging system (QuickGel 6200, Monad Biotech, China), amplification products that were of the correct size and displayed a single distinct band were selected for sequencing to confirm their identities.

### Allele-specific PCR (AS-PCR)

Allele-specific PCR (AS-PCR) was employed to amplify the target sequences, utilizing the identical reaction system applied in tick species molecular identification. The PCR reactions were carried out using a Veriti 96 Well Thermal Cycler (Applied Biosystems, Singapore). The cycling conditions for each mutation detection were as follows: for the C190A mutation, an initial denaturation occurred at 98 °C for 3 min, followed by 30 cycles of denaturation at 98 °C for 30 s, annealing at 53.5 °C for 1 min, extension at 72 °C for 20 s, and a final extension at 72 °C for 7 min. For the G215T mutation, an initial denaturation occurred at 96 °C for 2 min, followed by denaturation at 94 °C for 1 min, annealing at 52.9 °C for 1 min, extension at 72 °C for 20 s, and a final extension at 72 °C for 7 min. For the T2134A mutation, an initial denaturation occurred at 96 °C for 3 min, followed by 35 cycles of denaturation at 96 °C for 1 min, annealing at 54.7 °C for 1 min, extension at 72 °C for 20 s, and a final extension at 72 °C for 5 min. Two separate reactions were conducted on each sample to detect different genotypes (resistant or sensitive), with each reaction set containing two non-template controls (Table 1). The DL 500 bp DNA Marker and PCR products were subjected to electrophoresis on a 2% agarose gel and visualized using a gel imaging system.

**Table 1:** Primes for allele-specific PCR

Insecticide/gene	Mutation (amplicon size)	Primer sequence (forward and reverse (5′–3′))	Reference	Description
Pyrethroids/ *VGSC* domain II	C190A (101 bp)	GGAAAACCATCGGTGCTC	[44]	Domain II forward susceptible
		GGAAAACCATCGGTGCTA		Domain II forward resistant
		CTTCGTAGTTCTTGCCAAAG		Domain II common reverse
Pyrethroids/ *VGSC* domain II	G215T (92 bp)	CTTGACCTTTGTCCTGGG	[23]	Domain II forward susceptible
		CTTGACCTTTGTCCTGGT		Domain II forward resistant
		ACTTGTGTTTACTTTCTTCGTAGT		Domain II common reverse
Pyrethroids/ *VGSC* domain III	T2134A (68 bp)	TTATCTTCGGCTCCTTCT	[43]	Domain III forward susceptible
		TTATCTTCGGCTCCTTCA		Domain III forward resistant
		TTGTTCATTGAAATTGTCGA		Domain III common reverse

## Results

**Fig. 1:**
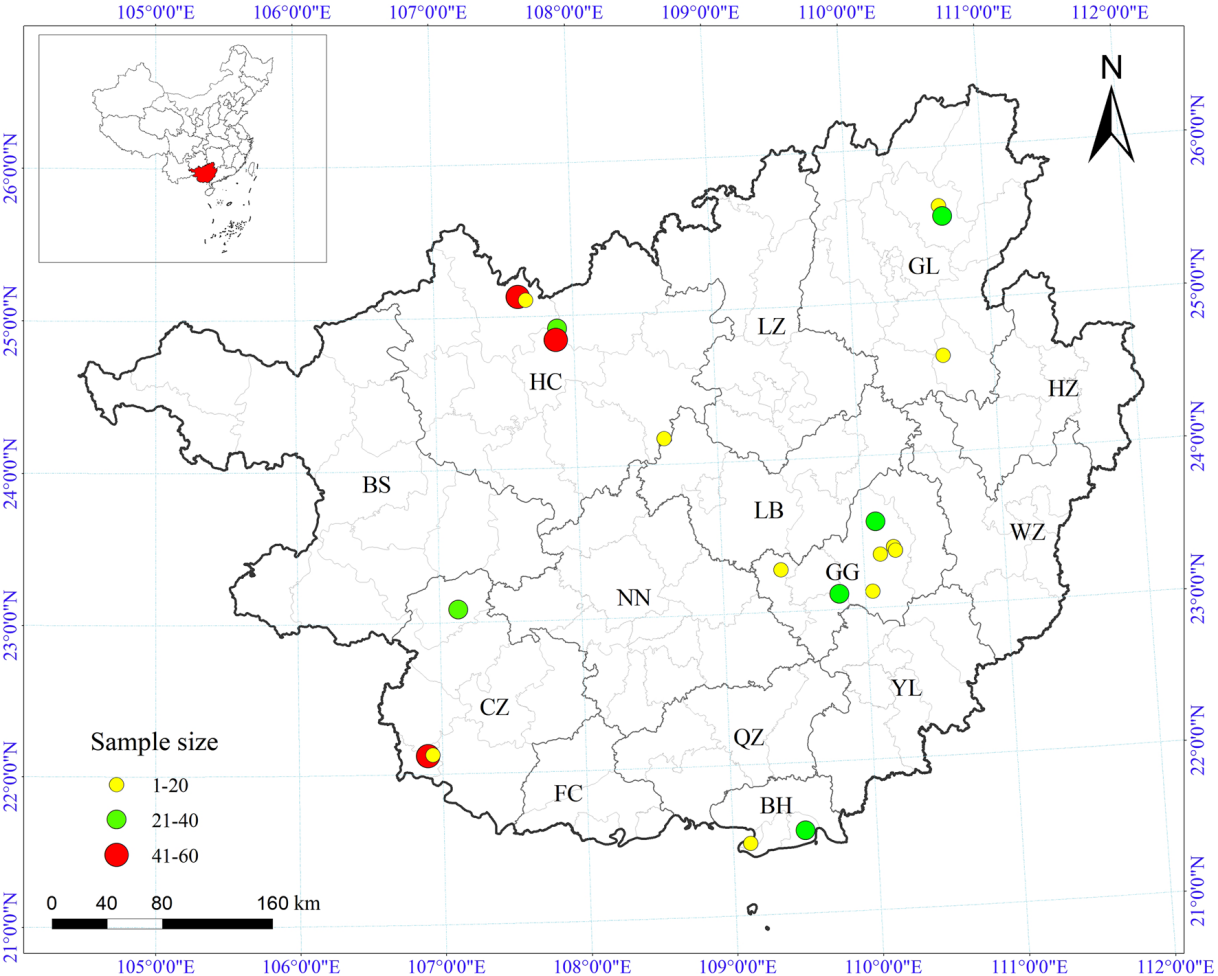
Map of tick sampling locations. The map shows the geographical locations of the five prefecture-level cities in the Guangxi Zhuang autonomous region involved in this study: BH (Beihai), CZ (Chongzuo), GG (Guigang), GL (Guilin), HC (Hechi). The size and color of the circles represent the sample volume at each sampling point

Based on morphological and molecular identification results, 447 tick samples collected from five prefecture-level cities in the Guangxi were unequivocally identified as *R. microplus* (Fig. 1). These samples underwent screening for mutations in the *VGSC* gene at mutation positions C190A, G215T, and T2134A. Overall, the mutation rate at these loci was relatively low. Specifically, the individual mutation rate was highest for C190A at 4.9% (22/447), followed by G215T at 4.0% (18/447) and T2134A at 1.3% (6/447). Four mutation types/combinations were identified across the samples, namely C190A, G215T, C190A + G215T, and T2134A (Table 2).

**Table 2:** Mutation types of *VGSC* genes in *R. microplus* in five cites in Guangxi, China

Population code ^a^	Number of ticks	Mutation types/combinations of *VGSC* genes			
		C190A	G215T	C190A + G215T	T2134A
HC	172	8.1% (14)	2.9%(5)	0.6% (1)	0.0% (0)
CZ	93	1.1% (1)	4.3% (4)	2.2% (2)	0.0% (0)
GG	97	0.0% (0)	2.1% (2)	0.0% (0)	6.2%(6)
GL	53	7.5% (4)	0.0% (0)	0.0% (0)	0.0% (0)
BH	32	0.0% (0)	12.5% (4)	0.0% (0)	0.0% (0)
Total	447	4.3% (19)	3.4% (15)	0.7% (3)	1.3%(6)

^a^Population codes as in Fig. 1

**Fig. 2:**
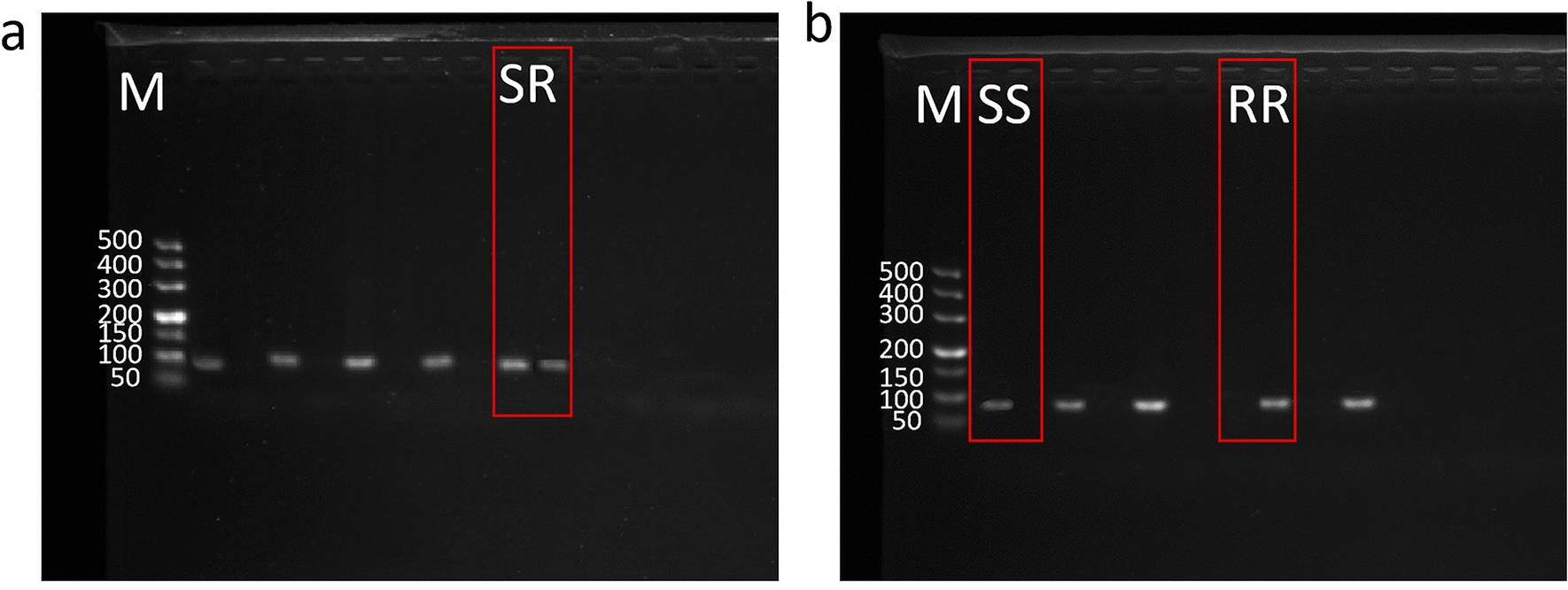
Allele-specific PCR detection of mutations in the *voltage-gated sodium channel* (*VGSC*) domain II in *R. microplus*. **a** Heterozygote and homozygote of T2134A mutation, **b** heterozygote and homozygote of G215T mutation; M, 500 bp DNA marker; SS, susceptible homozygote; SR, heterozygote; RR, resistant homozygote

In samples from Hechi and Chongzuo, three identical mutation types were observed, including C190A, G215T, and C190A + G215T. Specifically, in Hechi, the mutation frequencies were 8.1% (14/172) for C190A, 2.9% (5/172) for G215T, and 0.6% (1/172) for C190A + G215T. In Chongzuo, the frequencies for these three mutation types/combinations were 1.1% (1/93), 4.3% (4/93), and 2.2% (2/93), respectively. Although the same three mutation types were identified in both regions, C190A mutation predominated in Hechi, while G215T was predominant in Chongzuo (Fig. 2a). Furthermore, in the 97 *R. microplus* samples from Guigang, two mutation types were detected: T2134A (6.2%, 6/97) (Fig. 2b) and G215T (2.1%, 2/97). In contrast, samples from Beihai and Guilin each exhibited only one mutation type. Specifically, the mutation type and frequency in Beihai were G215T (12.5%, 4/32), while in Guilin, it was C190A (7.5%, 4/53).

**Fig. 3:**
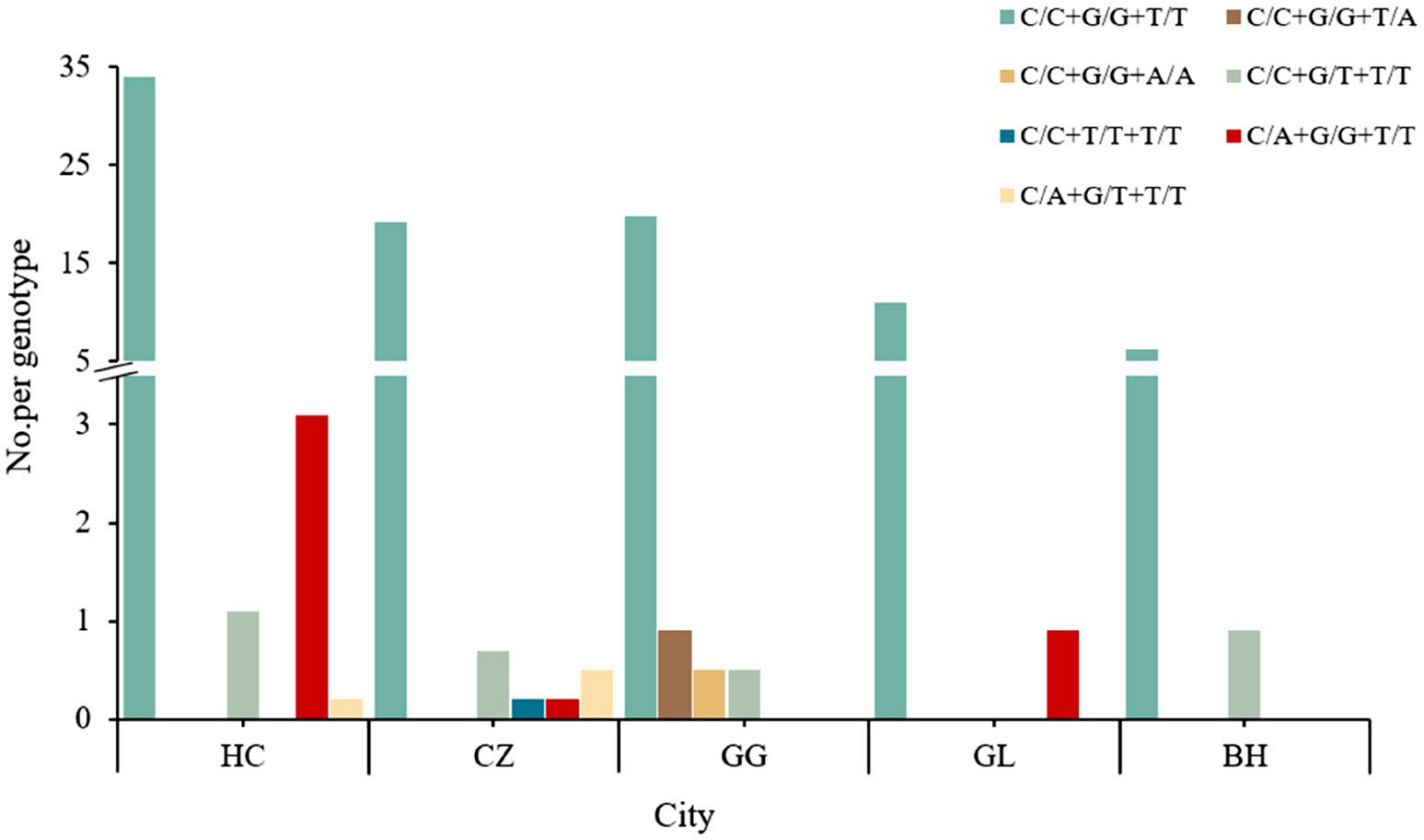
Frequency distribution of genotype combinations at C190A, G215T and T2134A sites of *VGSC* in *R. microplus*

Additionally, this study identified a total of seven genotypic combinations. The most prevalent combination was C/C + G/G + T/T, wherein no mutations occurred at any of the three sites, comprising 90.4% of the samples. Among the detected *VGSC* gene mutation types, C190A mutation was the most frequent, accounting for 4.3% (19/447), with only one genotype identified, C/A + G/G + T/T, at a proportion of 4.3% (Fig. 3). Conversely, the frequency of the C190A + G215T mutation combination was the lowest, with its sole genotype being C/A + G/T + T/T (0.7%). Additionally, two genotypes were detected for both G215T and T2134A mutation types. For G215T mutation, the two genotypes collectively accounted for 3.4%, with C/C + G/T + T/T being the dominant genotype at 93.3%. Similarly, two genotypes collectively represented 1.3% of the T2134A mutation type, with C/C + G/G + T/A being the predominant genotype at 66.7%.

## Discussion

The Guangxi Zhuang autonomous region lies within a subtropical monsoon climate zone, characterized by an average annual temperature ranging from 16.5 to 23.1 °C [27]. Abundant rainfall and the flourishing livestock and tourism industries contribute to the region’s rich diversity of tick species and elevated transmission risks. Among these species, *R. microplus* stands out due to its short lifecycle, high reproductive potential, and dominance in the area. Historically, chemical acaricides have been the primary means of controlling tick populations. However, their excessive use and misuse have led to the development of resistance among tick populations, resulting in ineffective control measures. This study collected 447 samples of *R. microplus* ticks from five prefecture-level cities located in different parts of Guangxi, ensuring a comprehensive and ample distribution of samples for objective research. Leveraging AS-PCR technology, the study investigated the presence of resistance-related mutations in the *VGSC* gene of *R. microplus* in Guangxi, elucidating the status of pyrethroid resistance in these ticks in the region.

Since the first report of synthetic pyrethroid resistance in *R. microplus* in Australia in 1989, there have been continuous reports of resistance to this class of acaricides globally [28–30]. Studies from different countries have shown that different mutations in the *VGSC* gene of *R. microplus* lead to different resistance phenotypes. Notably, the substitution of cytosine with adenine at nucleotide position 190 (C190A) in the domain II S4-5 linker region results in the substitution of leucine with isoleucine at amino acid position 64 (L64I). This mutation confers resistance to multiple pyrethroids, with resistance ratios exceeding 100 for cypermethrin and deltamethrin, and exceeding 400 for fenvalerate [9, 17]. In this study, the frequency of the C190A mutation in the 447 *R. microplus* from Guangxi was 4.9%, with the C/A genotype being predominant. This mutation was found in ticks from Hechi, Chongzuo, and Guilin. Studies in Brazil, Argentina, Australia, and South Africa have also identified the domain II C190A mutation associated with resistance to several synthetic pyrethroid compounds, with frequencies ranging from 38 to 100% in populations resistant to cypermethrin and deltamethrin [16, 19, 20, 31–33]. Additionally, the homozygous mutant genotype (A/A) was significantly correlated with survival rates under different doses of cypermethrin and deltamethrin [16, 31]. However, this mutation was not detected in ticks from Guigang to Beihai in this study, consistent with previous findings in southern India [34, 35].

In contrast, the nucleotide substitution at position 215 (G → T) in domain II of the *VGSC* gene (G215T) is closely adjacent to the C190A mutation site [18]. This mutation results in the substitution of valine (Val) with glycine (Gly) at amino acid position 72 (G72V) and is associated with resistance of *R. microplus* to flumethrin. A study conducted in Australia in 2010 sequenced the domain II S4-5 linker region of *VGSC* in *R. microplus* and found that the frequency of the homozygous mutant genotype of G215T in various populations was moderately correlated with survival rates at discriminative concentrations of flumethrin (*r* = 0.74) [18]. Notably, the study also observed a stronger relationship between the G215T mutation and resistance to cypermethrin (*r* = 0.93) when occurring simultaneously with the C190A mutation. In this study, the G215T mutation was detected in all areas except Guilin, with a frequency of 4.0%, and two genotypes (G/T and T/T) were simultaneously present. Furthermore, the frequency of the C190A + G215T combined mutation in *R. microplus* from all five regions of Guangxi was 0.7%, with the genotype being C/A + G/T. These findings suggest that there is interaction between the two mutations in the same gene, indicating that resistance to synthetic pyrethroids may be conferred by either the G215T mutation (G/T or T/T) or simultaneous mutations at positions 190 and 215. Combining these results with those of Jonsson et al. (2010) suggests that multiple mutations may have a synergistic effect, and combined mutations at different sites may be more effective than single mutations alone [18]. Therefore, timely monitoring of different mutation types and changes in resistance levels is crucial for controlling tick-borne diseases and the rapid development of insecticide resistance.

In addition to the C190A and G215T mutations in domain II of the *VGSC* gene, nucleotide mutation at position 2134 (T2134A) in the S6 segment of domain III was reported in the 1990s to be highly associated with resistance of *R. microplus* to synthetic pyrethroid acaricides [36]. The T2134A nucleotide mutation results in the substitution of phenylalanine (Phe) with isoleucine (Ile) at position 1550 (F1550I) in the protein sequence. Studies conducted in Mexico confirmed that this mutation was associated with resistance of local *R. microplus* populations to flumethrin, deltamethrin, and cypermethrin, and the mortality rate of ticks exposed to synthetic pyrethroids was correlated with the frequency of resistant alleles [37]. In 2011, Rodriguez-Vivas RI et al. found by AS-PCR that the resistance allele frequencies of five *R. microplus* populations increased from an initial 5–46% to 66–95% after 8–24 consecutive months of cypermethrin selective pressure, and that the frequency of resistance purists in the three populations exposed to cypermethrin was strongly correlated with larval survival, confirming that the T2134A mutation is associated with *R. microplus* resistance in Mexico [38]. In this study, compared with the C190A and G215T mutations, the frequency of the T2134A mutation was the lowest, accounting for only 1.3%. The results are consistent with those of a study conducted by Mendes et al. in São Paulo State, Brazil [39]. Although mutations in domain III lead to rapid development of resistance to synthetic pyrethroids [40], this mutation was not found in resistant *R. microplus* populations in Brazil [16, 41], Benin [42], Kerala [34], and Bihar [33]. Furthermore, this study revealed that *R. microplus* in Hechi, Beihai, Chongzuo, and Guilin did not exhibit the T2134A mutation, consistent with the results described by Guerrero et al. using AS-PCR [43].

While this study provides valuable theoretical insights into insecticide resistance management in *R. microplus*, there are still some limitations. Firstly, only 447 *R. microplus* samples from five regions in Guangxi were tested for mutations related to resistance to synthetic pyrethroid acaricides. The scope of this study was limited by the number of samples collected, the use of only one type of acaricide, and the geographic restriction to certain areas within Guangxi. Consequently, these limitations prevent a comprehensive evaluation of tick resistance to a variety of acaricides across the entire region. Given the rapid development of resistance, it will be necessary to conduct monitoring of resistance to common acaricides in other regions of Guangxi to provide comprehensive and systematic reference data for tick control in Guangxi. Secondly, this study did not identify the phenotypic resistance of ticks based on biological assays but relied on molecular detection of resistance using existing molecular markers. Additionally, AS-PCR techniques cannot detect metabolic enzyme-based resistance, and ticks would be considered susceptible if they did not possess the specific mutation that the AS-PCR was designed to detect [43]. Therefore, future research should conduct joint analysis of resistance-related single-nucleotide polymorphism (SNP) loci at the whole-genome level and combine phenotypic resistance to further clarify the characteristics of tick resistance mutations in Guangxi, ensuring the effectiveness of tick control strategies and mitigating the harm of ticks to animal husbandry and tourism in Guangxi.

## Conclusions

These findings indicate a relatively low frequency of *VGSC* gene mutations associated with pyrethroid resistance in *R. microplus* in Guangxi. Furthermore, there are variations in mutation types and genotypes across different regions. A comprehensive understanding of these mutations will facilitate accurate monitoring of tick resistance emergence and development in various regions, ensuring the effective implementation of tick control strategies.

## Data Availability

The data supporting the findings of the study are available within the article.
