# Correction: Molecular assessment of *voltage-gated sodium channel* (*VGSC*) gene mutations in *Rhipicephalus microplus* from Guangxi, China

**DOI:** 10.1186/s13071-024-06443-x

**Published:** 2024-08-22

**Authors:** Na Jiang, Ting Xie, Chunfu Li, Rui Ma, Ai Gao, Mengyun Liu, Shurong Wang, Qingan Zhou, Xiankai Wei, Jian Li, Wei Hu, Xinyu Feng

**Affiliations:** 1 College of Life Sciences, Inner Mongolia University, Hohhot, China; 2 Hechi Animal Disease Prevention and Control Center, Hechi, Guangxi China; 3 Guangxi Center for Animal Disease Control and Prevention, Nanning, Guangxi China; 4 Basic Medical College, Guangxi University of Chinese Medicine, Nanning, Guangxi China; 5 Department of Infectious Diseases, Huashan Hospital, State Key Laboratory of Genetic Engineering, Ministry of Education Key Laboratory for Biodiversity Science and Ecological Engineering, Ministry of Education Key Laboratory of Contemporary Anthropology, School of Life Sciences, Fudan University, Shanghai, China; 6 School of Global Health, Chinese Center for Tropical Diseases Research, Shanghai Jiao Tong University School of Medicine, Shanghai, China; 7 One Health Center, Shanghai Jiao Tong University-The University of Edinburgh, Shanghai, 20025 China

**Correction: Parasites & Vectors (2024) 17:307** 10.1186/s13071-024-06383-6

Following publication of the erratum, it came to the attention of the authors that an incorrect file had been provided for the graphical abstract. The graphical abstract has since been corrected with the correction file; please follow the link to the original article [1] to see the new, correct graphical abstract. The authors thank you for reading this erratum and apologize for any inconvenience caused.
